# Supplementary material for: Excess of Rare Missense Variants in Hearing Loss Genes in Sporadic Meniere Disease
Source: Front Genet. 2019 Feb 15;10:76. doi: 10.3389/fgene.2019.00076 (PMC6385525; doi:10.3389/fgene.2019.00076)
Supplement: Supplementary file 1 [file Data_Sheet_1.docx]

Supplementary Material

# Excess of rare missense variants in hearing loss genes in sporadic Meniere disease

Alvaro Gallego-Martinez^1^, Teresa Requena^1^, Pablo Roman-Naranjo^1^, Jose A. Lopez-Escamez^1,2*^ for the Meniere Disease Consortium (MeDiC)

*** Correspondence**

Dr. Jose A. Lopez-Escamez

Otology & Neurotology Group CTS 495, Department of Genomic Medicine, GENYO. Centre for Genomics and Oncological Research: Pfizer / University of Granada / Andalusian Regional Government, PTS Granada

Avenida de la Ilustracion, 114

18016 Granada SPAIN

E-mail: [antonio.lopezescamez@genyo.es](mailto:antonio.lopezescamez@genyo.es)

**Table S1. Design of the gene panel.** Regions included contain 50 bp upstream and 50 bp downstream of the entire genes selected. Mitochondrial genes were also included in this panel. However, their analysis were separated and not included in the present study.

| **Gene** | **Genome location** | **Exon** | **Length** | **Gene** | **Genome location** | **Exon** | **Length** |
| --- | --- | --- | --- | --- | --- | --- | --- |
|  | **(hg19)** | **Nº.** | **(kb)** |  | **(hg19)** | **Nº.** | **(kb)** |
|  |  |  |  |  |  |  |  |
| ACTG1 | chr17:79476947-79479942 | 3 | 2666 | **MT-ATP8** | chrM:8315-8621 | 1 | 307 |
| ADD1 | chr4:2845534-2931853 | 17 | 13391 | ESPN | chr1:6484798-6521480 | 14 | 5685 |
| ARNT2 | chr15:80696642-80890328 | 21 | 8996 | EYA4 | chr6:133561686-133853308 | 23 | 11034 |
| CCDC50 | chr3:191046816-191116509 | 11 | 10398 | FAM107B | chr10:14560506-14816946 | 21 | 9203 |
| CEACAM16 | chr19:45202371-45214036 | 7 | 2583 | FAM136A | chr2:70523057-70529272 | 2 | 3286 |
| CLDN14 | chr21:37832869-37948917 | 8 | 3653 | GJB2 | chr13:20761554-20767164 | 2 | 2709 |
| COCH | chr14:31343691-31364321 | 11 | 4442 | GRHL2 | chr8:102504610-102682004 | 17 | 7780 |
| DPT | chr1:168664645-168698552 | 4 | 2188 | KCNE1 | chr21:35818936-35884623 | 7 | 6481 |
| DTNA | chr18:32073204-32471858 | 30 | 13821 | KCNE3 | chr11:74165836-74178723 | 3 | 3707 |
| POU4F3 | chr5:145718537-145720133 | 2 | 1382 | KCNJ10 | chr1:160007207-160040101 | 2 | 5506 |
| WHRN | chr9:117164310-117267780 | 14 | 6942 | KCNQ1 | chr11:2466171-2870390 | 19 | 5756 |
| NR3B2 | chr14:76776907-76968228 | 15 | 5584 | KCNQ4 | chr1:41249634-41306174 | 16 | 5783 |
| **MT-CO2** | chrM:7535-8318 | 1 | 784 | MARVELD2 | chr5:68710889-68740207 | 8 | 5871 |
| **MT-TC** | chrM:10008-10453 | 1 | 446 | MICA | chr6:31367511-31384066 | 6 | 4618 |
| **MT-ND1** | chrM:3256-4311 | 1 | 1056 | MIF | chr22:24236141-24237464 | 2 | 1309 |
| **MT-ATP6** | chrM:8476-9256 | 1 | 781 | MSRB3 | chr12:65672373-65860737 | 10 | 6387 |
| **MT-TL1** | chrM:3179-3353 | 1 | 175 | MYH14 | chr19:50706835-50813852 | 43 | 11331 |
| **MT-TV** | chrM:1550-1718 | 1 | 169 | MYO7A | chr11:76839260-76926336 | 51 | 15511 |
| **MT-TI** | chrM:4212-4380 | 1 | 169 | NFKB1 | chr4:103422436-103538509 | 29 | 8102 |
| **MT-TQ** | chrM:4278-4449 | 1 | 172 | P2RX2 | chr12:133195316-133199022 | 7 | 2939 |
| **MT-TM** | chrM:4351-4518 | 1 | 168 | PNPT1 | chr2:55861148-55921095 | 24 | 7916 |
| **MT-TW** | chrM:5461-5628 | 1 | 168 | PRKCB | chr16:23847250-24231982 | 20 | 12059 |
| **MT-TA** | chrM:5536-5704 | 1 | 169 | RDX | chr11:110045555-110167497 | 19 | 7564 |
| **MT-TN** | chrM:5606-5778 | 1 | 173 | SEMA3D | chr7:84624819-84816221 | 20 | 10062 |
| **MT-ND3** | chrM:5710-5875 | 1 | 166 | SLC12A2 | chr5:127419408-127525430 | 27 | 13463 |
| **MT-TY** | chrM:5775-5940 | 1 | 166 | SLC26A4 | chr7:107301030-107358304 | 24 | 8300 |
| **MT-TS1** | chrM:7395-7563 | 1 | 169 | THAP1 | chr8:42691767-42698524 | 4 | 2868 |
| **MT-TD** | chrM:7467-7634 | 1 | 168 | TJP2 | chr9:71736130-71870174 | 25 | 9812 |
| **MT-TK** | chrM:8244-8413 | 1 | 170 | TLR10 | chr4:38773810-38784661 | 4 | 4617 |
| **MT-TG** | chrM:9940-10107 | 1 | 168 | TPRN | chr9:140086019-140098695 | 3 | 3277 |
| **MT-TR** | chrM:10354-10518 | 1 | 165 | TRIOBP | chr22:38092945-38172613 | 26 | 15061 |
| **MT-TH** | chrM:12087-12255 | 1 | 169 | USH1C | chr11:17515392-17566013 | 29 | 6576 |
| **MT-TS2** | chrM:12156-12314 | 1 | 159 | USH1G | chr17:72912126-72919408 | 3 | 3868 |
| **MT-TL2** | chrM:12215-12385 | 1 | 171 | WFS1 | chr4:6271526-6305042 | 8 | 5313 |
| **MT-TT** | chrM:15837-16002 | 1 | 166 |  |  |  |  |

**Table S2. References used to support the gene panel selection for MD panel**. DFNA, DFNB and others genes are separated in three tables.

| **Locus (OMIM)** | **Gene (OMIM)** | **Reference** |
| --- | --- | --- |
| [DFNA2A](http://www.ncbi.nlm.nih.gov/omim/600101) | [KCNQ4](http://www.ncbi.nlm.nih.gov/omim/603537) | [Kubisch et al., 1999](http://www.ncbi.nlm.nih.gov/entrez/query.fcgi?db=PubMed&cmd=Retrieve&list_uids=10025409&dopt=Abstract) |
| [DFNA3A](http://www.ncbi.nlm.nih.gov/omim/601544) | [GJB2](http://www.ncbi.nlm.nih.gov/omim/121011) | [Kelsell et al., 1997](http://www.ncbi.nlm.nih.gov/entrez/query.fcgi?db=PubMed&cmd=Retrieve&list_uids=9139825&dopt=Citation) |
| [DFNA4](http://www.ncbi.nlm.nih.gov/pubmed?term=21368133) | [MYH14](http://www.ncbi.nlm.nih.gov/omim/608568) | [Donaudy et al., 2004](http://www.ncbi.nlm.nih.gov/entrez/query.fcgi?cmd=Retrieve&db=PubMed&dopt=Abstract&list_uids=15015131) |
|  | [CEACAM16](http://www.ncbi.nlm.nih.gov/pubmed/21368133) | [Zheng et al., 2011](http://www.ncbi.nlm.nih.gov/pubmed/21368133) |
| [DFNA6/14/38](http://omim.org/entry/600965) | [WFS1](http://www.ncbi.nlm.nih.gov/omim/606201) | [Bespalova et al., 2001](http://www.ncbi.nlm.nih.gov/entrez/query.fcgi?cmd=Retrieve&db=PubMed&list_uids=11709537&dopt=Abstract) ;  [Young et al., 2001](http://www.ncbi.nlm.nih.gov/entrez/query.fcgi?cmd=Retrieve&db=PubMed&list_uids=11709538&dopt=Abstract) |
| [DFNA9](http://www.ncbi.nlm.nih.gov/omim/601369) | [COCH](http://www.ncbi.nlm.nih.gov/omim/603196) | [Robertson et al., 1998](http://www.ncbi.nlm.nih.gov/entrez/query.fcgi?db=PubMed&cmd=Retrieve&list_uids=9806553&dopt=Abstract) |
| [DFNA10](http://www.ncbi.nlm.nih.gov/omim/601316) | [EYA4](http://www.ncbi.nlm.nih.gov/omim/603550) | [Wayne et al., 2001](http://www.ncbi.nlm.nih.gov/entrez/query.fcgi?cmd=Retrieve&db=PubMed&list_uids=11159937&dopt=Abstract) |
| [DFNA11](http://www.ncbi.nlm.nih.gov/omim/601317) | [MYO7A](http://www.ncbi.nlm.nih.gov/omim/276903) | [Liu et al., 1997](http://www.ncbi.nlm.nih.gov/entrez/query.fcgi?db=PubMed&cmd=Retrieve&list_uids=9354784&dopt=Citation) |
| [DFNA15](http://www.ncbi.nlm.nih.gov/omim/602459) | [POU4F3](http://www.ncbi.nlm.nih.gov/omim/602460) | [Vahava et al., 1998](http://www.ncbi.nlm.nih.gov/entrez/query.fcgi?db=PubMed&cmd=Retrieve&list_uids=9506947&dopt=Citation) |
| [DFNA20/26](http://www.ncbi.nlm.nih.gov/omim/604717) | [ACTG1](http://www.ncbi.nlm.nih.gov/omim/102560) | [Zhu et al., 2003](http://www.ncbi.nlm.nih.gov/entrez/query.fcgi?cmd=Retrieve&db=PubMed&list_uids=13680526&dopt=Abstract) ;  [van Wijk et al., 2003](http://www.ncbi.nlm.nih.gov/entrez/query.fcgi?cmd=Retrieve&db=pubmed&dopt=Abstract&list_uids=14684684) |
| [DFNA28](http://www.ncbi.nlm.nih.gov/omim/608641) | [GRHL2](http://www.ncbi.nlm.nih.gov/omim/608576) | [Peters et al., 2002](http://www.ncbi.nlm.nih.gov/entrez/query.fcgi?cmd=Retrieve&db=PubMed&list_uids=12393799&dopt=Abstract) |
| [DFNA41](http://www.omim.org/entry/608224) | [P2RX2](http://www.omim.org/entry/600844) | [Yan et al., 2013](http://www.ncbi.nlm.nih.gov/pubmed/23345450) |
| [DFNA44](http://www.ncbi.nlm.nih.gov/omim/607453) | [CCDC50](http://www.ncbi.nlm.nih.gov/omim/611051) | [Modamio-Hoybjor et al., 2007](http://www.ncbi.nlm.nih.gov/pubmed/17503326?ordinalpos=1&itool=EntrezSystem2.PEntrez.Pubmed.Pubmed_ResultsPanel.Pubmed_RVDocSum) |
| [DFNA51](http://omim.org/entry/613558) | [TJP2](http://www.ncbi.nlm.nih.gov/omim/607709) | [Walsh et al., 2010](http://www.ncbi.nlm.nih.gov/pubmed/20602916) |

| **Locus (OMIM)** | **Gene (OMIM)** | **Reference (OMIM)** |
| --- | --- | --- |
| [DFNB1A](http://www.ncbi.nlm.nih.gov/entrez/dispomim.cgi?id=220290) | [GJB2](http://www.ncbi.nlm.nih.gov/omim/121011) | [Kelsell et al., 1997](http://www.ncbi.nlm.nih.gov/pubmed/9139825?dopt=Abstract) |
| [DFNB2](http://www.ncbi.nlm.nih.gov/omim/600060) | [MYO7A](http://www.ncbi.nlm.nih.gov/omim/276903) | [Liu et al., 1997](http://www.ncbi.nlm.nih.gov/pubmed/9171832?dopt=Citation) ;  [Weil et al., 1997](http://www.ncbi.nlm.nih.gov/pubmed/9171833?dopt=Citation) |
| [DFNB4](http://www.ncbi.nlm.nih.gov/omim/600791) | [SLC26A4](http://www.ncbi.nlm.nih.gov/omim/605646) | [Li et al., 1998](http://www.ncbi.nlm.nih.gov/pubmed/9500541?dopt=Abstract) |
| [DFNB18](http://www.ncbi.nlm.nih.gov/omim/602092) | [USH1C](http://www.ncbi.nlm.nih.gov/omim/605242) | [Ouyang et al., 2002](http://www.ncbi.nlm.nih.gov/pubmed/12136232?dopt=Abstract) ; [Ahmed et al., 2002](http://www.ncbi.nlm.nih.gov/pubmed/12107438?dopt=Abstract) |
| [DFNB24](http://www.ncbi.nlm.nih.gov/omim/611022) | [RDX](http://www.ncbi.nlm.nih.gov/omim/179410) | [Khan et al., 2007](http://www.ncbi.nlm.nih.gov/pubmed/17226784?ordinalpos=2&itool=EntrezSystem2.PEntrez.Pubmed.Pubmed_ResultsPanel.Pubmed_RVDocSum) |
| [DFNB28](http://www.ncbi.nlm.nih.gov/omim/609823) | [TRIOBP](http://www.ncbi.nlm.nih.gov/omim/609761) | [Shahin et al., 2006](http://www.ncbi.nlm.nih.gov/pubmed/16385458?dopt=Abstract) ; [Riazuddin et al., 2006](http://www.ncbi.nlm.nih.gov/pubmed/16385457?dopt=Abstract) |
| [DFNB29](http://www.ncbi.nlm.nih.gov/omim/605608) | [CLDN14](http://www.ncbi.nlm.nih.gov/omim/605608) | [Wilcox et al., 2001](http://www.ncbi.nlm.nih.gov/pubmed/11163249?dopt=Abstract) |
| [DFNB31](http://www.ncbi.nlm.nih.gov/omim/607084) | [WHRN](http://www.ncbi.nlm.nih.gov/omim/607928) | [Mburu et al., 2003](http://www.ncbi.nlm.nih.gov/pubmed/12833159?dopt=Abstract) |
| [DFNB35](http://www.ncbi.nlm.nih.gov/omim/608565) | [ESRRB](http://www.ncbi.nlm.nih.gov/omim/602167) | [Collin et al., 2008](http://www.ncbi.nlm.nih.gov/pubmed/18179891?dopt=Abstract) |
| [DFNB36](http://www.ncbi.nlm.nih.gov/omim/609006) | [ESPN](http://www.ncbi.nlm.nih.gov/omim/606351) | [Naz et al., 2004](http://www.ncbi.nlm.nih.gov/pubmed/15286153?dopt=Abstract) |
| [DFNB49](http://www.ncbi.nlm.nih.gov/omim/610153) | [MARVELD2](http://www.ncbi.nlm.nih.gov/omim/610572) | [Riazuddin et al., 2006](http://www.ncbi.nlm.nih.gov/pubmed/17186462?ordinalpos=2&itool=EntrezSystem2.PEntrez.Pubmed.Pubmed_ResultsPanel.Pubmed_RVDocSum) |
| [DFNB70](http://omim.org/entry/614934?search=DFNB70&highlight=dfnb70) | [PNPT1](http://omim.org/entry/610316?search=PNPT1&highlight=pnpt1) | [von Ameln et al., 2012](http://www.ncbi.nlm.nih.gov/pubmed/23084290) |
| [DFNB74](http://www.ncbi.nlm.nih.gov/omim/613718) | [MSRB3](http://www.ncbi.nlm.nih.gov/omim/613719) | [Waryah et al., 2009](http://www.ncbi.nlm.nih.gov/pubmed/19650862) ; [Ahmed et al., 2011](http://www.ncbi.nlm.nih.gov/pubmed/21185009) |
| [DFNB79](http://omim.org/entry/613307) | [TPRN](http://www.ncbi.nlm.nih.gov/omim/613354) | [Rehman et al., 2010](http://www.ncbi.nlm.nih.gov/pubmed/20170899) ; [Li et al., 2010](http://www.ncbi.nlm.nih.gov/pubmed/20170898) |
|  | [ESRP1](https://omim.org/entry/609245) | [Rohacek et al., 2017](http://hereditaryhearingloss.org/29107558) |
|  |  |  |
|  | **Gene (OMIM)** | **Reference (OMIM)** |
|  | [ADD1](https://www.omim.org/entry/102680) | [Teggi et al., 2088](https://www.ncbi.nlm.nih.gov/pubmed/18667944) |
|  | [ARNT2](https://www.omim.org/entry/606036) | Own unpublished data |
|  | [DPT](https://www.omim.org/entry/125597) | Martin-Sierra C [et](https://www.ncbi.nlm.nih.gov/pubmed/27876815) al., 2017 |
|  | [DTNA](https://www.omim.org/entry/601239) | Requena T et al., [2015](https://www.ncbi.nlm.nih.gov/pubmed/25305078) |
|  | NR3B2 | [Chen J. et al., 2007](https://www.ncbi.nlm.nih.gov/pubmed/17765677) |
|  | FAM107B | [Scoles D et al., 2017](https://www.nature.com/articles/nature22044) |
|  | [FAM136A](https://www.omim.org/entry/616275) | Requena T et al., [2015](https://www.ncbi.nlm.nih.gov/pubmed/25305078) |
|  | [KCNE1](https://www.omim.org/entry/176261) | [Abbott GW 2016](https://www.ncbi.nlm.nih.gov/pubmed/26410412) |
|  | [KCNE3](https://www.omim.org/entry/604433) | [Abbott GW 2016](https://www.ncbi.nlm.nih.gov/pubmed/26410412) |
|  | [KCNJ10](https://www.omim.org/entry/602208) | [Smith RJH, 1998](https://www.ncbi.nlm.nih.gov/pubmed/20301640) |
|  | [KCNQ1](https://www.omim.org/entry/607542) | [Splawski I et al., 1997](https://www.ncbi.nlm.nih.gov/pubmed/9164812) |
|  | [MICA](https://www.omim.org/entry/600169) | [Gazquez I et al., 2012](https://www.ncbi.nlm.nih.gov/pubmed/22222578) |
|  | [MIF](https://www.omim.org/entry/153620) | [Gazquez I et al., 2013](https://www.ncbi.nlm.nih.gov/pubmed/23179933) |
|  | [PRKCB](https://www.omim.org/entry/176970) | [Martin-Sierra C et al., 2016](https://www.ncbi.nlm.nih.gov/pubmed/27329761) |
|  | [SEMA3D](https://www.omim.org/entry/609907) | [Martín-Sierra et al, 2017](https://www.ncbi.nlm.nih.gov/pubmed/27876815) |
|  | [SLC12A2](https://www.omim.org/entry/600840) | [Dixon MJ et al., 1999](https://www.ncbi.nlm.nih.gov/pubmed/10401008) |
|  | [THAP1](https://www.omim.org/entry/609520) | Own unpublished data |
|  | [TLR10](https://www.omim.org/entry/606270) | [Requena T et al., 2013](https://www.ncbi.nlm.nih.gov/pubmed/23370977) |
|  | [NFKB1](https://www.omim.org/entry/164011) | [Cabrera S et al., 2014](https://www.ncbi.nlm.nih.gov/pubmed/25397881) |
|  | [USH1G](https://www.omim.org/entry/607696) | [Miyasaka Y et al. 2016](https://www.ncbi.nlm.nih.gov/pubmed/26936824) |

**Table S3. Novel and rare variants in patients with sporadic MD**. List of rare variants validated through Sanger sequencing in MD patients. DbSNP accession numbers are detailed for each tested variant. Number of individuals with the variant in our MD cohort are described between parenthesis.

| **Gene** | **Location** | **dbSNP** | **MAF (gnomAD)** |  | **Case Freq (n)** |  |
| --- | --- | --- | --- | --- | --- | --- |
| *ESRRB* | 14:76957891  14:76966336  14:76966347 | rs201344770  rs200237229  rs201448899 | 0.0002096  0.0004445  0.0006266 |  | 0.0068181 (6)  0.0034090 (3)  0.0079545 (7) |  |
| *MARVELD2* | 5:68715821 | rs369265136 | 0.0000040 |  | 0.0034090 (3) |  |
| *SLC26A4* | 7:107336408 | rs200511789 | 0.0003572 |  | 0.0034090 (3) |  |
| *USH1G* | 17:72915919 | rs151242039 | 0.0006846 |  | 0.0034090 (3) |  |
|  | 17:72916543 | rs111033465 | 0.0003479 |  | 0.0010752 (1) |  |
| *GJB2* | 13:20763264  13:20763612  13:20763452  13:20763642  13:20763633 | rs111033186  rs72474224  rs80338945  rs2274084  rs374625633 | 0.007274  0.006587  0.0008818  0.04538  0.0000057 |  | 0.0079545 (7)  0.0068181 (6)  0.0034090 (3)  0.0118279 (11)  0.0010752 (1) |  |

**Table S4. Mean coverage percentage per gene region in ExAC, gnomAD and our MD panel.** Only the 18 genes with significant excess of missense variants in the Spanish population from Table 4 are detailed***.***

|  | **MEAN COVERAGE PERCENTAGE** | | |
| --- | --- | --- | --- |
| **GENE** | **ExAC** | **gnomAD** | **MD panel** |
| ***GJB2*** | 70.49 | 82.3 | 79.56 |
| ***ESRRB*** | 49.55 | 72.47 | 69.38 |
| ***CLDN14*** | 52.71 | 74.71 | 76.95 |
| ***USH1G*** | 56.66 | 71.04 | 53.53 |
| ***SLC26A4*** | 64.16 | 76.55 | 71.41 |
| ***MYH14*** | 28.94 | 48.89 | 46.44 |
| ***SEMA3D*** | 60.53 | 56.44 | 44.48 |
| ***NFKB1*** | 61.76 | 57.32 | 80.97 |
| ***CCDC50*** | 54.61 | 68.64 | 61.9 |
| ***P2RX2*** | 52.84 | 57.25 | 74.36 |
| ***FAM136A*** | 60.35 | 56.13 | 82.4 |
| ***RDX*** | 55.01 | 64.22 | 84.33 |
| ***TPRN*** | 29.88 | 47.96 | 64.23 |
| ***ESPN*** | 27.79 | 48.8 | 65.01 |
| ***SLC12A2*** | 56.83 | 48.99 | 74.8 |
| ***PRKCB*** | 65.12 | 58.28 | 71.61 |
| ***ADD1*** | 69.31 | 65.04 | 71.74 |

**Table S5. Selection of missense variants found in excess in the MD Spanish cohort.**

| Chr | Pos | Ref | Alt | Func.refGene | Gene.refGene | ExonicFunc.refGene | ExAC MAF | ExAC NFE MAF |
| --- | --- | --- | --- | --- | --- | --- | --- | --- |
| chr1 | 6488328 | C | T | exonic | ESPN | nonsynonymous SNV | 0.0005 | 0.0004 |
| chr1 | 6511753 | C | T | exonic | ESPN | nonsynonymous SNV | 0.0002 | 0.0003 |
| chr2 | 70524494 | A | C | exonic | FAM136A | nonsynonymous SNV | 8.24E-03 | 1.50E-02 |
| chr3 | 1.91E+08 | A | G | exonic | CCDC50 | nonsynonymous SNV | 0.0065 | 0.0050 |
| chr4 | 2900221 | A | G | exonic | ADD1 | nonsynonymous SNV | 8.24E-03 | 0 |
| chr4 | 1.04E+08 | C | A | exonic | NFKB1 | nonsynonymous SNV | 9.99E-02 | 6.03E-02 |
| chr4 | 1.04E+08 | A | G | exonic | NFKB1 | nonsynonymous SNV | 0.0097 | 0.0084 |
| chr4 | 1.04E+08 | G | T | exonic | NFKB1 | nonsynonymous SNV | 0.0019 | 0.0028 |
| chr5 | 1.27E+08 | A | T | exonic | SLC12A2 | nonsynonymous SNV | 1.66e-05 | 0 |
| chr5 | 1.46E+08 | C | G | exonic | POU4F3 | nonsynonymous SNV | 0.0004 | 0.0005 |
| chr7 | 84636125 | C | T | exonic | SEMA3D | nonsynonymous SNV | 0.0002 | 0.0002 |
| chr7 | 84727240 | A | G | exonic | SEMA3D | nonsynonymous SNV | 0.0172 | 0.0235 |
| chr7 | 1.07E+08 | A | G | exonic | SLC26A4 | nonsynonymous SNV | 4.13e-05 | 0 |
| chr7 | 1.07E+08 | T | C | exonic | SLC26A4 | nonsynonymous SNV | 0.0005 | 0.0006 |
| chr7 | 1.07E+08 | T | C | exonic | SLC26A4 | nonsynonymous SNV | 0.0083 | 0.0075 |
| chr7 | 1.07E+08 | T | G | exonic | SLC26A4 | nonsynonymous SNV | 0.0132 | 0.0005 |
| chr7 | 1.07E+08 | G | A | exonic | SLC26A4 | nonsynonymous SNV | 0.0044 | 0.0002 |
| chr7 | 1.07E+08 | C | T | exonic | SLC26A4 | nonsynonymous SNV | 0.0020 | 0.0029 |
| chr9 | 1.4E+08 | G | A | exonic | TPRN | nonsynonymous SNV | 0.0022 | 0.0036 |
| chr9 | 1.4E+08 | A | G | exonic | TPRN | nonsynonymous SNV | 0.0070 | 0.0004 |
| chr9 | 1.4E+08 | G | C | exonic | TPRN | nonsynonymous SNV | 0.0037 | 0.0002 |
| chr11 | 1.1E+08 | G | A | exonic | RDX | nonsynonymous SNV | 0.0023 | 0.0038 |
| chr11 | 1.1E+08 | G | C | exonic | RDX | nonsynonymous SNV | 2.48E-02 | 4.52e-05 |
| chr12 | 1.33E+08 | G | A | exonic | P2RX2 | nonsynonymous SNV | 0.0015 | 0.0024 |
| chr12 | 1.33E+08 | A | C | exonic | P2RX2 | nonsynonymous SNV | 0.0028 | 0.0029 |
| chr12 | 1.33E+08 | C | T | exonic | P2RX2 | nonsynonymous SNV | 0.0018 | 0.0003 |
| chr13 | 20763264 | C | T | exonic | GJB2 | nonsynonymous SNV | 0.0106 | 0.0045 |
| chr13 | 20763452 | A | G | exonic | GJB2 | nonsynonymous SNV | 0.0009 | 0.0015 |
| chr13 | 20763612 | C | T | exonic | GJB2 | nonsynonymous SNV | 0.0066 | 0.0019 |
| chr13 | 20763642 | C | T | exonic | GJB2 | nonsynonymous SNV | 0.0454 | 0.0023 |
| chr13 | 20763686 | C | A | exonic | GJB2 | nonsynonymous SNV | 5.01E-02 | 0 |
| chr13 | 20763710 | C | T | exonic | GJB2 | nonsynonymous SNV | 0.0004 | 0 |
| chr14 | 76957891 | G | A | exonic | ESRRB | nonsynonymous SNV | 0.0003 | 0.0001 |
| chr14 | 76966275 | T | C | exonic | ESRRB | nonsynonymous SNV | 0.0035 | 0.0050 |
| chr14 | 76966347 | C | T | exonic | ESRRB | nonsynonymous SNV | 0.0010 | 0.0014 |
| chr16 | 24046832 | C | G | exonic | PRKCB | nonsynonymous SNV | 0.0003 | 0.0005 |
| chr17 | 72915919 | C | T | exonic | USH1G | nonsynonymous SNV | 0.0006 | 0.0007 |
| chr17 | 72916365 | C | T | exonic | USH1G | nonsynonymous SNV | 0.0023 | 0.0018 |
| chr17 | 72916507 | C | T | exonic | USH1G | nonsynonymous SNV | 0.0118 | 0.0004 |
| chr17 | 72916543 | T | C | exonic | USH1G | nonsynonymous SNV | 0.0117 | 0.0003 |
| chr17 | 72916621 | T | C | exonic | USH1G | nonsynonymous SNV | 0.0001 | 0.0002 |
| chr19 | 50720992 | G | A | exonic | MYH14 | nonsynonymous SNV | 0.0002 | 0.0003 |
| chr19 | 50766628 | C | T | exonic | MYH14 | nonsynonymous SNV | 0.0005 | 0.0008 |
| chr19 | 50770231 | G | A | exonic | MYH14 | nonsynonymous SNV | 0.0041 | 0.0022 |
| chr21 | 37833809 | T | C | exonic | CLDN14 | nonsynonymous SNV | 0.0004 | 0.0007 |
| chr21 | 37833983 | G | A | exonic | CLDN14 | nonsynonymous SNV | 0.0403 | 0.0256 |

**Table S6. Average recombination rates (cM/Mb) in deCODE genetic maps for the chosen genes.** Only *USH1G* genomic location recombination rate seems to be notably higher than the human average recombination rate (1.2).

| **RR (Avg cM/Mb)** | |
| --- | --- |
| ***ESRRB*** | 1.8 |
| ***GJB2*** | 0.6 |
| ***USH1G*** | 2.9 |
| ***CLDN14*** | 1.4 |
| ***SLC26A4*** | 1.5 |

**Table S7. Linkage disequilibrium and pairwise correlations (R^2^) between pairs of missense variants reported in 1KGenomes populations for the genes with higher excess of missense variants in the Spanish cohort against global 1KGenomes population.**

| ***ESRRB*** | **rs61744548** | **rs201344770** | **rs553650212** | **rs188462546** | **rs201726554** | **rs201448899** |  |  |
| --- | --- | --- | --- | --- | --- | --- | --- | --- |
| **rs61744548** | 1 | 0 | 0 | 0 | 0 | 0 |  |  |
| **rs201344770** | 0 | 1 | 0 | 0 | 0 | 0 |  |  |
| **rs553650212** | 0 | 0 | 1 | 0 | 0 | 0 |  |  |
| **rs188462546** | 0 | 0 | 0 | 1 | 0 | 0 |  |  |
| **rs201726554** | 0 | 0 | 0 | 0 | 1 | 0 |  |  |
| **rs201448899** | 0 | 0 | 0 | 0 | 0 | 1 |  |  |
|  |  |  |  |  |  |  |  |  |
| ***GJB2*** | **rs111033186** | **rs111033218** | **rs72474224** | **rs2274084** | **rs111033222** |  |  |  |
| **rs111033186** | 1 | 0 | 0 | 0.001 | 0 |  |  |  |
| **rs111033218** | 0 | 1 | 0 | 0 | 0 |  |  |  |
| **rs72474224** | 0 | 0 | 1 | 0.001 | 0 |  |  |  |
| **rs2274084** | 0.001 | 0 | 0.001 | 1 | 0 |  |  |  |
| **rs111033222** | 0 | 0 | 0 | 0 | 1 |  |  |  |
|  |  |  |  |  |  |  |  |  |
| ***USH1G*** | **rs151242039** | **rs149002004** | **rs201644674** | **rs141688757** | **rs111033466** | **rs111033465** |  |  |
| **rs151242039** | 1 | 0 | 0 | 0 | 0 | 0 |  |  |
| **rs149002004** | 0 | 1 | 0 | 0 | 0 | 0 |  |  |
| **rs201644674** | 0 | 0 | 1 | 0 | 0 | 0 |  |  |
| **rs141688757** | 0 | 0 | 0 | 1 | 0 | 0 |  |  |
| **rs111033466** | 0 | 0 | 0 | 0 | 1 | 1 |  |  |
| **rs111033465** | 0 | 0 | 0 | 0 | 1 | 1 |  |  |
|  |  |  |  |  |  |  |  |  |
| ***CLDN14*** | **rs61745291** | **rs139437157** | **rs113350364** | **rs148223897** | **rs146395322** | **rs113831133** |  |  |
| **rs61745291** | 1 | 0 | 0 | 0 | 0 | 0.001 |  |  |
| **rs139437157** | 0 | 1 | 0 | 0 | 0 | 0 |  |  |
| **rs113350364** | 0 | 0 | 1 | 0 | 0 | 0 |  |  |
| **rs148223897** | 0 | 0 | 0 | 1 | 0 | 0 |  |  |
| **rs146395322** | 0 | 0 | 0 | 0 | 1 | 0 |  |  |
| **rs113831133** | 0.001 | 0 | 0 | 0 | 0 | 1 |  |  |
|  |  |  |  |  |  |  |  |  |
| ***SLC26A4*** | **rs200431470** | **rs111033243** | **rs200511789** | **rs55638457** | **rs17154335** | **rs17154347** | **rs17154353** | **rs111033255** |
| **rs200431470** | 1 | 0 | 0 | 0.046 | 0 | 0 | 0 | 0 |
| **rs111033243** | 0 | 1 | 0 | 0 | 0 | 0 | 0 | 0 |
| **rs200511789** | 0 | 0 | 1 | 0 | 0 | 0 | 0 | 0 |
| **rs55638457** | 0.046 | 0 | 0 | 1 | 0 | 0 | 0 | 0 |
| **rs17154335** | 0 | 0 | 0 | 0 | 1 | 0.412 | 0.309 | 0 |
| **rs17154347** | 0 | 0 | 0 | 0 | 0.412 | 1 | 0.749 | 0 |
| **rs17154353** | 0 | 0 | 0 | 0 | 0.309 | 0.749 | 1 | 0 |
| **rs111033255** | 0 | 0 | 0 | 0 | 0 | 0 | 0 | 1 |


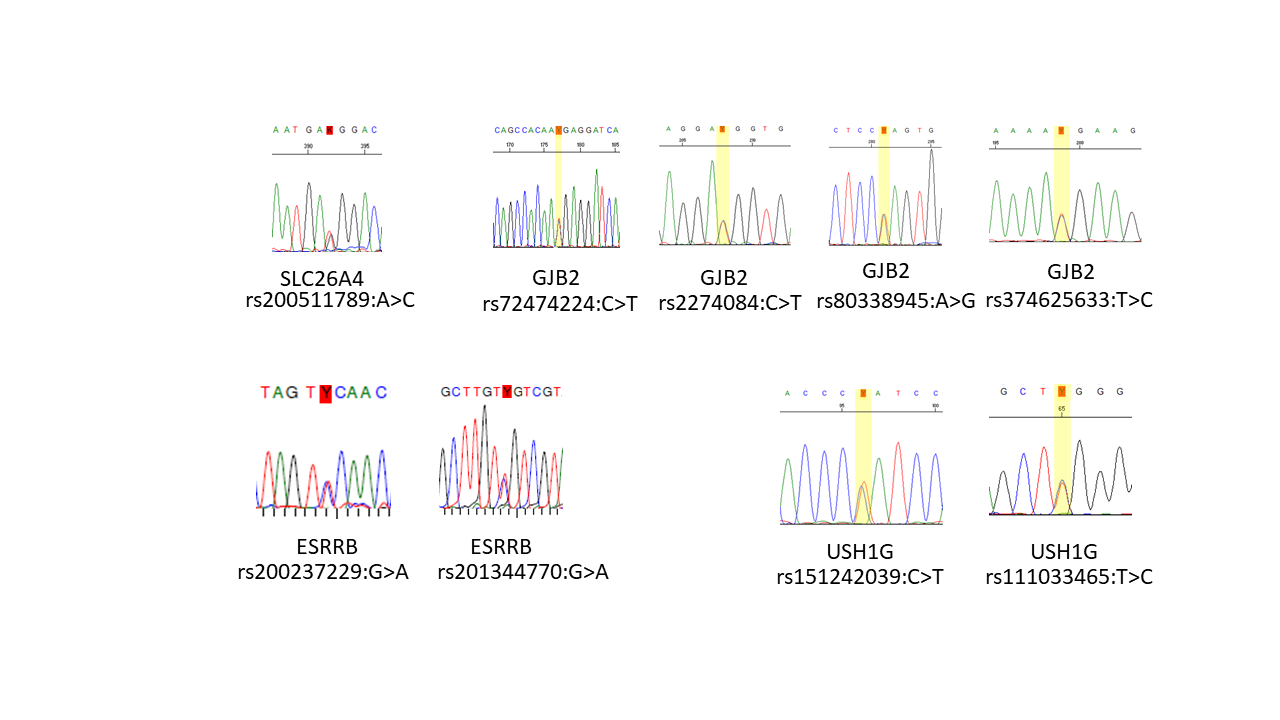
**Figure S1. Chromatographs for validated SNV through Sanger sequencing**.
